# Inverse correlation of intact PTH, oxidized PTH as well as non-oxidized PTH with 25-hydroxyvitamin D3 in kidney transplant recipients

**DOI:** 10.3389/fendo.2023.1178166

**Published:** 2023-05-31

**Authors:** Jiao Zuo, Ahmed A. Hasan, Carl-Friedrich Hocher, Philipp Kalk, Burkhard Kleuser, Bernhard K. Krämer, Berthold Hocher

**Affiliations:** ^1^ Department of Nephrology, Charité - Universitätsmedizin Berlin, Berlin, Germany; ^2^ Fifth Department of Medicine (Nephrology/Endocrinology/Rheumatology, Pneumonology), University Medical Center Mannheim, University of Heidelberg, Mannheim, Germany; ^3^ Institute of Pharmacy, Freie Universität Berlin, Berlin, Germany; ^4^ Klinik für Innere Medizin, Bundeswehrkrankenhaus Berlin, Berlin, Germany; ^5^ Diaverum Renal Care Center, Diaverum MVZ Am Neuen Garten Standort Ludwigsfelde, Potsdam, Germany; ^6^ European Center for Angioscience ECAS, Medical Faculty Mannheim of the University of Heidelberg, Mannheim, Germany; ^7^ Reproductive, Genetic Hospital of CITIC-Xiangya, Changsha, China; ^8^ Institute of Medical Diagnostics, IMD, Berlin, Germany

**Keywords:** 25-hydroxyvitamin D, 1,25-dihydroxyvitamin D, parathyroid hormone, intact parathyroid hormone, oxidized parathyroid hormone, non-oxidized parathyroid hormone

## Abstract

**Background:**

25-hydroxyvitamin D (25(OH)D) and potentially also 1,25-dihydroxyvitamin D (1,25(OH)2D) inhibits the synthesis of parathyroid hormone (PTH) in the chief cells of the parathyroid gland. Clinical studies showing a negative correlation between (25(OH)D and PTH are in good agreement with these findings in basic science studies. However, PTH was measured in these studies with the currently clinically used 2nd or 3rd generation intact PTH (iPTH) assay systems. iPTH assays cannot distinguish between oxidized forms of PTH and non-oxidized PTH. Oxidized forms of PTH are the by far most abundant form of PTH in the circulation of patients with impaired kidney function. Oxidation of PTH causes a loss of function of PTH. Given that the clinical studies done so far were performed with an PTH assay systems that mainly detect oxidized forms of PTH, the real relationship between bioactive non-oxidized PTH and 25(OH)D as well as 1,25(OH)2D is still unknown.

**Methods:**

To address this topic, we compared for the first time the relationship between 25(OH)D as well as 1,25(OH)2D and iPTH, oxPTH as well as fully bioactive n-oxPTH in 531 stable kidney transplant recipients in the central clinical laboratories of the Charité. Samples were assessed either directly (iPTH) or after oxPTH (n-oxPTH) was removed using a column that used anti-human oxPTH monoclonal antibodies, a monoclonal rat/mouse parathyroid hormone antibody (MAB) was immobilized onto a column with 500 liters of plasma samples. Spearman correlation analysis and Multivariate linear regression were used to evaluate the correlations between the variables.

**Results:**

There was an inverse correlation between 25(OH)D and all forms of PTH, including oxPTH (iPTH: r=-0.197, p<0.0001; oxPTH: r=-0.203, p<0.0001; n-oxPTH: r=-0.146, p=0.001). No significant correlation was observed between 1,25(OH)2D and all forms of PTH. Multiple linear regression analysis considering age, PTH (iPTH, oxPTH and n-oxPTH), serum calcium, serum phosphor, serum creatinine, fibroblast growth factor 23 (FGF23), osteoprotegerin (OPG), albumin, and sclerostin as confounding factors confirmed these findings. Subgroup analysis showed that our results are not affected by sex and age.

**Conclusion:**

In our study, all forms of PTH are inversely correlated with 25-hydroxyvitamin D (25(OH)D). This finding would be in line with an inhibition of the synthesis of all forms of PTH (bioactive n-oxPTH and oxidized forms of PTH with minor or no bioactivity) in the chief cells of the parathyroid glad.

## 1 Introduction

The parathyroid polypeptide hormone (PTH) is synthesized and cleaved into its active form in the parathyroid glands. PTH is vital for calcium homeostasis, and it has various end-organ targets by which it can regulate the calcium levels, such as the bones, kidneys, and intestines. Calcium levels in the blood act as a negative feedback loop, signaling the parathyroid glands to stop releasing PTH (1). *In vitro* and *in vivo*, FGF23 inhibits PTH mRNA production and secretion in an alpha klotho (KL)-dependent way (2). The PTH hormone increases calcium levels when serum calcium concentration is low (1, 3, 4). Then, intracellular calcium concentrations increase, inhibiting exocytosis of PTH *via* signal transduction across the CaSR (5).

PTH’s active form contains 84 amino acids as a single-chain polypeptide hormone (5). There are two methionine residues in PTH, located at positions 8 and 18. Oxidation of these residues inhibits or eliminates PTH’s biological function (6–8). After oxidation they are separated into four forms (1) n-oxPTH; (2) PTH oxidized at methionine residue 8 (ox (Met8)PTH); (3) ox (Met18)PTH; (4) PTH oxidized at both residues 8 and 18 (ox (Met8,18)PTH). The bioactivity degree from high to low was: n-oxPTH, ox (Met18)PTH, ox (Met8)PTH, and ox (Met8,18)PTH (6). In CKD stage 2-4 children, 90% of iPTH is oxidized (oxPTH); in adult CKD patients on dialysis, 89.5% of iPTH is oxidized (oxPTH), and in kidney transplant patients, 89% of iPTH is oxidized (oxPTH) (9).

Vitamin D is present in a variety of forms in the blood. The two forms in the liver and kidneys are 25-hydroxyvitamin D (25 (OH)D) and 1,25-dihydroxyvitamin D (1,25(OH)2D). Vitamin D status can be assessed by measuring the body’s distribution of 25(OH) D in the blood (10–13). Circulating blood levels of 1,25 (OH)2 D are much lower than those of 1,25 (OH)2 D (14). Vitamin D is mainly synthesized in the kidney (as well as in pregnant women’s placentas) and is circulated in the bloodstream. As a consequence, 1,25 (OH)2 vitamin D concentrations are dependent on both the supply of 25(OH) vitamin D and the kidney fraction (15–17). 25 (OH)D has the most prolonged half-life (2 to 3 weeks), while 1,25(OH)2D has a short half-life (1-3 days) (18). The serum concentrations of 1,25(OH)2D are around 0.1% of that of 25(OH)D concentrations. PTH, hypocalcemia, and hypophosphatemia result in the induction of CYP27B1 in the kidney, which produces 1-alpha-hydroxylation, resulting in higher concentrations of 1,25(OH)2D (5, 19). Through secondary hyperparathyroidism, an inadequate supply of substrate 25(OH)D will stimulate the renal CYP27B1-hydroxylase to maintain or increase the production of 1,25(OH)2D metabolites, whereas a deficiency of substrate for the extrarenal CYP27B1-hydroxylase results in a decrease in 1,25(OH)2D products (20). The vitamin D receptor (VDR) on the parathyroid gland stimulates 1,25(OH)2D to inhibit PTH directly (21).

Nowadays, most published articles focus on the PTH and 25(OH)D in different diseases or populations (22–24). The relationship between PTH and vitamin D in kidney transplant patients is poorly studied, specifically analyzing 25(OH)D and 1,25(OH)2D simultaneously. In this study, we analyzed the relationship between different statuses of vitamin D and the relationship of PTH forms (iPTH, oxPTH, and n-oxPTH) with 25(OH)D, 1,25(OH)2D, serum calcium, serum phosphor, creatinine, FGF23, Osteoprotegerin (OPG), Alkaline phosphatase (ALP), and sclerostin. Moreover, we studied the relationship between iPTH, oxPTH, and n-oxPTH with 25(OH)D, 1,25(OH)2D in two subgroups (female/male and age above 50 years/below 50 years).

## 2 Materials and methods

### 2.1 Study population

**Figure 1:**
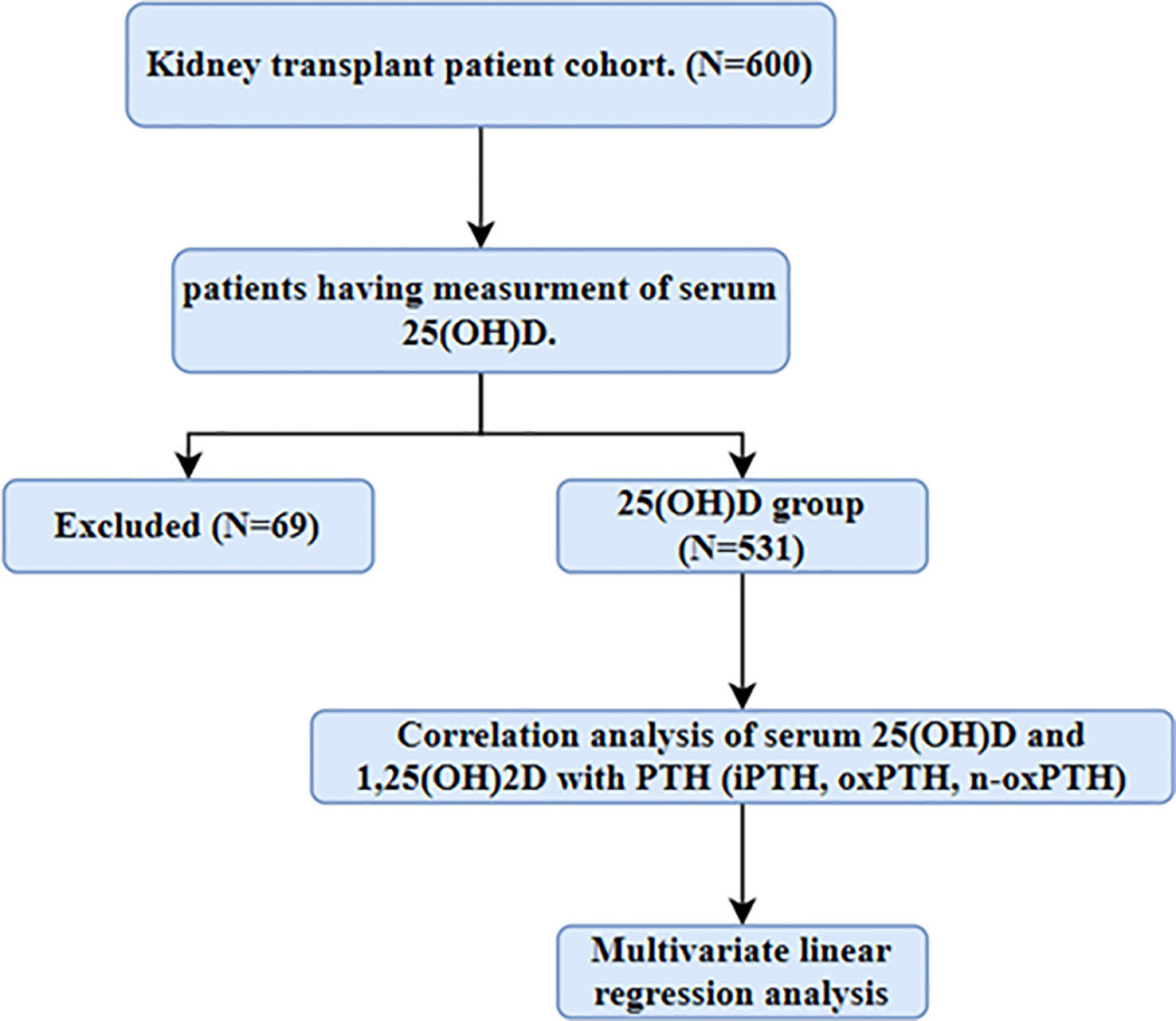
Flow-chart of study population. Patients who have measurement of serum 25(OH)D was included in this study.

A total of 531 of the 600 kidney transplant patients undergoing routine check-up in the transplant outpatient clinic Charité-Mitte, Berlin, Germany, had 25VD levels and a functional graft were included in this study cohort (**Figure 1**). The exclusion criteria are patients with an active infection, malignancy, acute rejection, recent cardiovascular events, or those unwilling to participate. Local ethics committees approved this study, and we obtained informed consent from all study participants.

Samples of patients’ blood and urine were collected routinely from April 2012 until December 2012. Those samples were kept frozen at −80°C. Baseline data, i.e., post-transplant duration, cold ischemia time, age, and gender of the recipient, were collected from patients’ records. In addition, clinical parameters such as 25-hydroxyvitamin D, 1,25-dihydroxy vitamin D, iPTH, oxPTH, n-oxPTH, total cholesterol, calcium, and phosphorus were all analyzed in the central clinical laboratories of the Charité.

### 2.2 Assays

Second and third-generation PTH assays (also known as intact PTH assays), which are now utilized in clinical practice, cannot distinguish between oxPTH and n-oxPTH. Both oxPTH and n-oxPTH are measured (iPTH = n-oxPTH + oxPTH). Samples were assessed either directly (iPTH) or after oxPTH (n-oxPTH) was removed using a column that used anti-human oxPTH monoclonal antibodies, a monoclonal rat/mouse parathyroid hormone antibody (MAB) was immobilized onto a column with 500 liters of plasma samples. At room temperature, the columns were mixed end-over-end for 2 h and then washed with 250 l of ammonium acetate buffer pH 7.0 and, after washing with 250 l of ammonium acetate buffer pH 7.0 containing 20% acetonitrile, 2 times with elution buffer (0.05 M formic acid, pH 3.5). Separate fractions of the flow-through, wash fraction, and eluate were lyophilized. Reconstituted samples were then analyzed by Roche Intact PTH assay with 500 l of PBS buffer, pH 7.4 (25). The commercially available ELISA used to measure cFGF23 was the FGF23 (C-terminal) multi-matrix ELISA with catalog number BI-20702, produced by Biomedica in Austria, according to the manufacturer’s instructions (https://www.bmgrp.com/wp-content/uploads/2019/03/bi-20702-fgf23-elisa-validation-data-150306.pdf). Sclerostin and OPG were measured using commercially available kits (Catalogue BI-20492, and BI-20403, Biomedica Medizinprodukte GmbH, Vienna, Austria) according to the instructions of the manufacturer as recently described (26–28). Apart from PTH isoforms, FGF23, OPG and sclerostin, all clinical laboratory parameters were examined in the university hospital Charité’s central clinical laboratories and subjected to routine quality checks according to German law.

### 2.3 Statistical analysis

SPSS version 23.0 (IBM corporation, New York, USA) was used for statistical evaluation. The mean was used to represent all continuous variable parameters. To assess the correlation between variables, we used Spearman correlation analysis. The Multivariate linear regression was performed using iPTH, oxPTH, and n-oxPTH as the dependent variables. Parameters recognized in the literature as affecting PTH concentration (age, serum calcium, serum phosphor, creatinine, FGF23, OPG, ALP, and sclerostin) were introduced into a multivariate linear regression model. Statistically significant differences were considered as *p*<0.05. All figures were made by GraphPad Prism 8 (GraphPad Software Corporation, California, USA) and presented as mean ± SEM.

## 3 Results

### 3.1 Characteristics of the study population

**Table 1** shows the characteristics of the study population. In this study cohort, the mean age was 54.74 years, and there were 202 females and 329 of males. The frequency distributions of 25(OH)D, 1,25(OH)2D, iPTH, oxPTH, and n-oxPTH showed an approximately normal distribution (**Supplementary Figures 1A, B**).

**Table 1:** Characteristics of the study population (N=531).

Parameters	Mean ± SD	N
Age	54.74 ± 14.51	531
Sex (Male/Female)	329/202	531
Dialysis before transplant (months)	49.30 ± 35.73	531
Cold ischemia time (hours)	10.20 ± 7.37	531
25(OH)D (ng/mL)	52.81 ± 28.77	531
1,25(OH)2D (pg/mL)	90.03 ± 43.58	461
iPTH (pg/mL)	96.17 ± 70.04	513
ox PTH (pg/mL)	87.25 ± 64.50	513
n-ox PTH (pg/mL)	10.27 ± 6.76	513
Calcium_Serum (mmol/L)	2.47 ± 0.19	529
Phosphor_Serum (mmol/L)	0.89 ± 0.25	525
HbA1c (%)	5.88 ± 0.85	402
Creatinine (mg/dL)	1.73 ± 0.69	531
FGF23 (pg/mL)	6.03 ± 18.58	490
OPG (pmol/I)	4.90 ± 2.50	531
ALP (IU/L)	82.84 ± 36.24	529
Sclerostin (pg/mL)	50.61 ± 24.16	531
TC (mg/dL)	223.49 ± 53.56	529
TG (mg/dL)	225.22 ± 181.58	529
CRP (mg/L)	3.11 ± 3.77	209

25(OH)D, 25-hydroxy-vitamin D; 1,25(OH)2D, 1,25-dihydroxyvitamin D; iPTH, intact parathyroid hormone; oxPTH, oxidized parathyroid hormone; n-oxPTH, non-oxidized parathyroid hormone; HbA1c, hemoglobin A1c; FGF23, Fibroblast Growth Factor 23 receptor; OPG, osteoprotegerin; ALP, alkaline phosphatase; TC, total cholesterol; TG, triglyceride; CRP, C-reactive protein.

### 3.2 Correlation of serum 25(OH)D with 1,25(OH)2D; correlation of iPTH with oxPTH, and n-oxPTH

**Figure 2:**
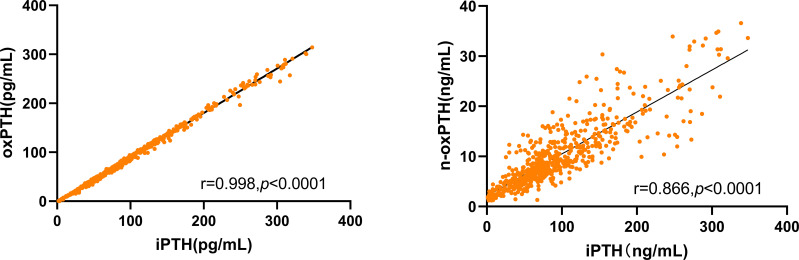
Correlation of oxPTH and n-oxPTH with iPTH in 531 kidney transplant patients. oxPTH and n-oxPTH were both significant related with iPTH in our study.

In transplant patients, 25(OH)D was correlated with 1,25(OH)2D (r=0.120, *p*=0.009) (**Supplementary Figure 2**). OxPTH had a strong relationship with iPTH (r=0.998, *p*<0.0001), in comparison, n-oxPTH was not that correlated with iPTH (r=0.866, *p*<0.0001) (**Figure 2**).

### 3.3 Correlation of serum 25(OH)D with clinical chemistry parameters

**Figure 3:**
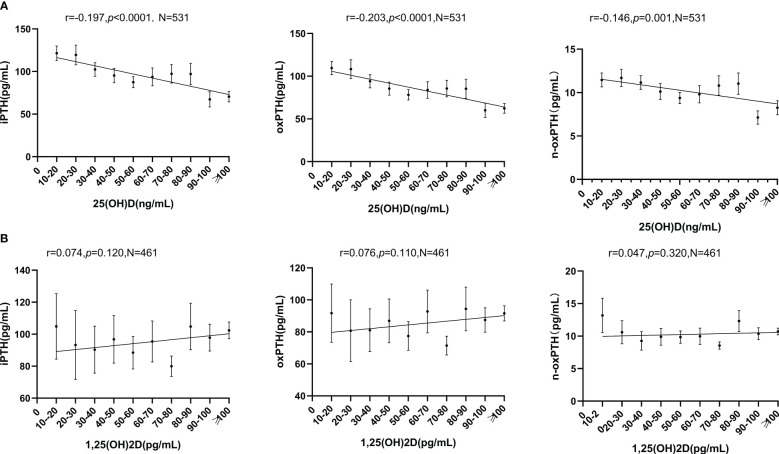
**(A)** Relationship between 25(OH)D and PTH (iPTH, oxPTH, n-oxPTH). All forms of PTH were significant related with 25(OH)D. Spearman correlation analysis was used. Graphs were presented as mean ± SEM. **(B)** Relationship between 1,25(OH)2D and PTH (iPTH, oxPTH, n-oxPTH). All forms of PTH were not related with 1,25(OH)2D. Spearman correlation analysis was used. Graphs were presented as mean ± SEM.

25(OH)D correlated with iPTH (r=-0.197, *p*<0.0001); oxPTH (r=-0.203, *p*<0.0001), n-oxPTH (r=-0.146, *p*=0.001) (**Table 2**, **Figure 3A**). According to the concentration of 25(OH)D, the study cohort was divided into 11 groups: (0-10ng/ml; 10-20ng/ml; 20-30ng/ml; 30-40ng/ml; 40-50ng/ml; 50-60ng/ml; 60-70ng/ml; 70-80ng/ml; 80-90ng/ml; 90-100ng/ml; ≥100ng/ml), and the mean ± SEM of PTH (iPTH, oxPTH and n-oxPTH) in each group was taken for graphing. There were negative correlations across the sexes (iPTH, female: r=-0.265, *p*<0.001; male: r=-0.147, *p*=0.009); (oxPTH, female: r=-0.258, *p*<0.001; male: r=-0.160, *p*=0.004) (n-oxPTH, female: r=-0.272, *p*<0.001; male: r=-0.056, *p*=0.316) (**Supplementary Figure 3A**); and different stage of age (iPTH, age <50 years: r=-0.162, *p*=0.026; age ≥50 years: r=-0.234, *p*<0.0001); (oxPTH, age <50 years: r=-0.172, *p*=0.018; age ≥50 years: r=-0.235, *p*<0.0001); (n-oxPTH, age <50 years: r=-0.086, *p*=0.241; age ≥50 years: r=-0.187, *p*=0.001) (**Supplementary Figure 4A**). Furthermore, the multivariate linear regression for 25(OH)D considering age, PTH (iPTH, oxPTH and n-oxPTH), serum calcium, serum phosphor, serum creatinine, FGF23, OPG, ALP and sclerostin showed that 25(OH)D was significantly correlated with iPTH (unstandardized coefficients B: -0.094, *p*=53×10^-6^) (**Table 3A**); oxPTH (unstandardized coefficients B: -0.108, *p*=2×10^-5^) (**Table 3B**); and n-oxPTH (unstandardized coefficients B: -0.561, *p*=0.015) (**Table 3C**)

**Table 2:** Correlation of serum 25(OH)D and 1,25(OH)2D with clinical chemistry parameters.

Parameters		25(OH)D			1,25(OH)2D	
	r	*P*	N	r	*P*	N
Age	-0.015	0.732	531	-0.033	0.475	461
Cold ischemia time (hours)	0.014	0.758	513	0.132	0.005	445
iPTH (pg/mL)	-0.197	<0.0001	513	0.074	0.120	444
ox PTH (pg/mL)	-0.203	<0.0001	513	0.076	0.110	444
n-ox PTH (pg/mL)	-0.146	0.001	513	0.047	0.320	445
FGF23(pg/mL)	-0.015	0.739	490	-0.402	<0.0001	423
Calcium_Serum (mmol/L)	-0.106	0.015	529	0.096	0.039	459
Phosphor_Serum (mmol/L)	-0.017	0.694	525	-0.274	<0.0001	455
HbA1c (%)	-0.059	0.239	402	-0.031	0.572	346
Creatinine (mg/dL)	-0.001	0.977	531	-0.302	<0.0001	461
OPG (pmol/I)	-0.041	0.347	531	-0.068	0.143	461
ALP(IU/L)	-0.182	<0.0001	529	0.125	0.007	459
Sclerostin (pg/mL)	0.044	0.316	531	-0.150	0.001	461
TC (mg/dL)	0.068	0.120	529	-0.062	0.185	459
TG (mg/dL)	0.038	0.383	529	-0.020	0.675	459
CRP (mg/L)	-0.063	0.367	209	-0.144	0.080	149

25(OH)D, 25-hydroxy-vitamin D; 1,25(OH)2D, 1,25-dihydroxyvitamin D; iPTH, intact parathyroid hormone; oxPTH, oxidized parathyroid hormone; n-oxPTH, non-oxidized parathyroid hormone; HbA1c, hemoglobin A1c; FGF23, Fibroblast Growth Factor 23 receptor; OPG, osteoprotegerin; ALP, alkaline phosphatase; TC, total cholesterol; TG, triglyceride; CRP, C-reactive protein.

The Spearman correlation analysis was performed.

**Table 3A:** Multivariate linear regression for iPTH.

Independent variable		25(OH)D	95%CI	Independent variable		1,25(OH)2D	
	*p* value	Coefficients B			*p* value	Coefficients B	95%CI
Constant	21×10^-6^	88.333	47.957~128.709		0.005	87.398	25.874~148.922
Age	0.166	0.141	-0.059~0.341		0.806	0.040	-0.279~0.359
iPTH	53×10^-6^	-0.094	-0.139~-0.049		0.243	0.043	-0.029~0.114
Serum Calcium	0.212	-9.443	-24.275~5.389		0.195	14.980	-7.732~37.692
Serum Phosphor	0.118	-9.597	-21.640~2.445		0.153	-13.775	-32.712~5.161
Serum Creatinine	0.169	3.352	-1.431~8.134		0.000	-16.582	-24.505~-8.660
FGF23	0.205	0.109	-0.060~0.279		0.024	-0.296	-0.553~-0.039
OPG	0.038	-1.295	-2.516~-0.074		0.799	-0.255	-2.219~1.709
ALP	0.647	-0.017	-0.091~0.056		0.035	0.130	0.009~0.251
Sclerostin	0.717	-0.019	-0.124~0.086		0.107	-0.130	-0.289~0.028

25(OH)D, 25-hydroxy-vitamin D; 1,25(OH)2D, 1,25-dihydroxyvitamin D; iPTH, intact parathyroid hormone; FGF23, Fibroblast Growth Factor 23 receptor; OPG, osteoprotegerin; ALP, alkaline phosphatase.

Coefficients B: unstandardized coefficients B.

95%CI: 95% Confidence interval for B.

Parameters recognized in the literature as affecting PTH concentration (age, serum calcium, serum phosphor, creatinine, FGF23, OPG, ALP, and sclerostin) were introduced into the multivariate linear regression model.

**Table 3B:** Multivariate linear regression for oxPTH.

Independent variable		25(OH)D		Independent variable		1,25(OH)2D	
	*p* value	Coefficients B	95%CI		*p* value	Coefficients B	95%CI
Constant	24×10^-6^	87.451	47.190~127.712		0.006	86.929	25.342~148.516
Age	0.157	0.144	-0.056~0.343		0.812	0.039	-0.280~0.358
oxPTH	20×10^-6^	-0.108	-0.157~-0.059		0.234	0.047	-0.031~0.126
Serum Calcium	0.226	-9.117	-23.900~5.666		0.191	15.144	-7.583~37.870
Serum Phosphor	0.117	-9.593	-21.608~2.421		0.163	-13.481	-32.421~5.460
Serum Creatinine	0.141	3.577	-1.192~8.346		0.000	-16.558	-24.480~-8.636
FGF23	0.234	0.102	-0.066~0.271		0.024	-0.295	-0.552~-0.039
OPG	0.035	-1.311	-2.529~-0.094		0.782	-0.276	-2.243~1.690
ALP	0.738	-0.012	-0.086~0.061		0.032	0.133	0.012~0.254
Sclerostin	0.614	-0.027	-0.132~0.078		0.098	-0.134	-0.293~0.025

25(OH)D, 25-hydroxy-vitamin D; 1,25(OH)2D, 1,25-dihydroxyvitamin D; oxPTH, oxidized parathyroid hormone; FGF23, Fibroblast Growth Factor 23 receptor; OPG, osteoprotegerin; ALP, alkaline phosphatase.

Coefficients B: unstandardized coefficients B.

95%CI: 95% Confidence interval for B.

Parameters recognized in the literature as affecting PTH concentration (age, serum calcium, serum phosphor, creatinine, FGF23, OPG, ALP, and sclerostin) were introduced into the multivariate linear regression model.

**Table 3C:** Multivariate linear regression for n-oxPTH.

Independent variable		25(OH)D		Independent variable		1,25(OH)2D	
	*p* value	Coefficients B	95%CI		*p* value	Coefficients B	95%CI
Constant	40×10^-7^	96.681	56.223~137.139		0.004	88.079	27.942~150.215
Age	0.370	0.091	-0.108~0.291		0.681	0.066	-0.250~0.383
n-oxPTH	0.015	-0.561	-1.013~-0.109		0.368	0.319	-0.378~1.016
Serum Calcium	0.078	-13.312	-28.108~1.484		0.250	13.152	-9.273~35.578
Serum Phosphor	0.455	-4.495	-16.305~7.314		0.118	-14.711	-33.179~3.757
Serum Creatinine	0.375	2.132	-2.584~6.849		0.000	-15.070	-22.847~-7.293
FGF23	0.164	0.122	-0.050~0.293		0.016	-0.317	-0.575~-0.060
OPG	0.041	-1.288	-2.520~-0.055		0.838	-0.204	-2.175~1.766
ALP	0.206	-0.048	-0.122~0.026		0.019	0.147	0.024~0.270
Sclerostin	0.913	-0.006	-0.111~0.100		0.068	-0.148	-0.306~0.011

25(OH)D, 25-hydroxy-vitamin D; 1,25(OH)2D, 1,25-dihydroxyvitamin; n-oxPTH, non-oxidized parathyroid hormone; FGF23, Fibroblast Growth Factor 23 receptor; OPG, osteoprotegerin; ALP, alkaline phosphatase.

Coefficients B: unstandardized coefficients B.

95%CI: 95% Confidence interval for B.

Parameters recognized in the literature as affecting PTH concentration (age, serum calcium, serum phosphor, creatinine, FGF23, OPG, ALP, and sclerostin) were introduced into the multivariate linear regression model.

### 3.4 Correlation of serum 1,25(OH)2D with clinical chemistry parameters

There are some differences between 1,25(OH)2D and PTH (iPTH, oxPTH and n-oxPTH): no correlation with iPTH (r=0.074, *p*=0.120); oxPTH (r=0.076, *p*=0.110); n-oxPTH (r=0.047, *p*=0.320) (**Table 2**, **Figure 3B**), but 1,25(OH)2D correlated with PTH in females (iPTH: r=0.198, *p*=0.01; oxPTH: r=0.198, *p*=0.01; n-oxPTH: r=0.160, *p*=0.036) (**Supplementary Figure 3B**); as well as in age <50 years subgroup (iPTH: r=0.179, *p*=0.022; oxPTH: r=0.178, *p*=0.023; n-oxPTH: r=0.162, *p*=0.038) (**Supplementary Figure 4B**). 1,25(OH)2D was not correlated with iPTH, oxPTH and n-oxPTH in male (**Supplementary Figure 3B**) and age ≥50 years (**Supplementary Figure 4B**). When serum 25(OH)D≥30ng/mL, 1,25(OH)2D was significantly correlated with iPTH (r=0.119, *p*=0.030) and oxPTH (r=0.120, *p*=0.027) (**Supplementary Figure 5**). Multiple linear regression analysis performed in different concentrations of 25(OH)D (<30ng/ml and≥30ng/ml) confirmed these findings (**Tables 4A**–**C**).

**Table 4A:** Multivariate linear regression for iPTH in vitamin D sufficient/deficient group.

Independent variable		25(OH)D≥30ng/mL		Independent variable		25(OH)D<30ng/mL	
	*p* value	Coefficients B	95%CI		*p* value	Coefficients B	95%CI
Constant	10×10^-6^	92.179	51.850~132.507		0.646	-4.639	-24.680~15.402
Age	0.552	-0.066	-0.282~0.151		0.092	0.076	-0.013~0.164
1,25(OH)2D	0.005	0.093	0.028~0.158		0.265	0.020	-0.015~0.054
iPTH	0.026	-0.056	-0.105~-0.007		0.934	0.001	-0.022~0.024
Serum Calcium	0.178	-10.382	-25.502~4.739		0.167	4.810	-2.056~11.677
Serum Phosphor	0.483	4.387	-7.897~16.671		0.142	5.345	-1.835~12.525
Serum Creatinine	0.941	-0.197	-5.441~5.047		0.887	-0.217	-3.238~2.804
FGF23	0.006	0.276	0.080~0.473		0.244	0.034	-0.024~0.092
OPG	0.274	-0.821	-2.296~0.654		0.242	-0.262	-0.705~0.181
ALP	0.585	-0.022	-0.103~0.058		0.493	-0.013	-0.051~0.025
Sclerostin	0.296	-0.053	-0.154~0.047		0.717	0.011	-0.050~0.072

Vitamin D sufficient group: 25(OH)D≥30ng/mL; vitamin D deficient group: 25(OH)D<30ng/mL.

25(OH)D, 25-hydroxy-vitamin D; 1,25(OH)2D, 1,25-dihydroxyvitamin D; iPTH, intact parathyroid hormone; FGF23, Fibroblast Growth Factor 23 receptor; OPG, osteoprotegerin; ALP, alkaline phosphatas.

Coefficients B: unstandardized coefficients B.

95%CI: 95% Confidence interval for B.

Parameters recognized in the literature as affecting PTH concentration (age, serum calcium, serum phosphor, creatinine, FGF23, OPG, ALP, and sclerostin) were introduced into the multivariate linear regression model.

**Table 4B:** Multivariate linear regression for oxPTH in vitamin D sufficient/deficient group.

Independent variable		25(OH)D≥30ng/mL		Independent variable		25(OH)D<30ng/mL	
	*p* value	Coefficients B	95%CI		*p* value	Coefficients B	95%CI
Constant	10×10^-6^	92.083	51.786~132.380		0.647	-4.641	-24.776~15.494
Age	0.555	-0.065	-0.281~0.152		0.096	0.075	-0.014~0.165
1,25(OH)2D	0.005	0.094	0.029~0.158		0.278	0.019	-0.016~0.054
oxPTH	0.020	-0.064	-0.118~-0.010		0.935	0.001	-0.024~0.026
Serum Calcium	0.179	-10.349	-25.455~4.756		0.168	4.824	-2.073~11.722
Serum Phosphor	0.498	4.232	-8.033~16.496		0.140	5.414	-1.815~12.643
Serum Creatinine	0.966	-0.112	-5.350~5.125		0.907	-0.180	-3.233~2.874
FGF23	0.006	0.273	0.078~0.469		0.254	0.033	-0.025~0.091
OPG	0.278	-0.813	-2.287~0.661		0.236	-0.266	-0.711~0.178
ALP	0.584	-0.022	-0.102~0.058		0.533	-0.012	-0.051~0.026
Sclerostin	0.291	-0.054	-0.154~0.046		0.797	0.008	-0.054~0.071

Vitamin D sufficient group: 25(OH)D≥30ng/mL; vitamin D deficient group: 25(OH)D < 30ng/mL.

25(OH)D, 25-hydroxy-vitamin D; 1,25(OH)2D, 1,25-dihydroxyvitamin D; iPTH, intact parathyroid hormone; FGF23, Fibroblast Growth Factor 23 receptor; OPG, osteoprotegerin; ALP, alkaline phosphatas.

Coefficients B: unstandardized coefficients B.

95%CI: 95% Confidence interval for B.

Parameters recognized in the literature as affecting PTH concentration (age, serum calcium, serum phosphor, creatinine, FGF23, OPG, ALP, and sclerostin) were introduced into the multivariate linear regression model.

**Table 4C.** Multivariate linear regression for n-oxPTH in vitamin D sufficient/deficient group.

Independent variable		25(OH)D≥30ng/mL		Independent variable		25(OH)D<30ng/mL	
	*p* value	Coefficients B	95%CI		*p* value	Coefficients B	95%CI
Constant	20×10^-7^	98.211	58.073~138.350		0.730	-3.611	-24.404~17.183
Age	0.464	-0.080	-0.296~0.135		0.074	0.079	-0.008~0.166
1,25(OH)2D	0.007	0.089	0.024~0.153		0.303	0.019	-0.018~0.056
n-oxPTH	0.147	-0.340	-0.799~0.120		0.987	-0.002	-0.237~0.233
Serum Calcium	0.085	-13.024	-27.872~1.823		0.209	4.544	-2.607~11.696
Serum Phosphor	0.240	7.140	-4.795~19.074		0.287	3.801	-3.265~10.866
Serum Creatinine	0.798	-0.665	-5.787~4.457		0.946	0.102	-2.877~3.082
FGF23	0.006	0.277	0.079~0.474		0.250	0.035	-0.025~0.095
OPG	0.280	-0.8111	-2.287~0.665		0.249	-0.271	-0.735~0.193
ALP	0.250	-0.049	-0.131~0.034		0.864	-0.003	-0.043~0.036
Sclerostin	0.374	-0.045	-0.145~0.055		0.827	0.007	-0.057~0.072

Vitamin D sufficient group: 25(OH)D≥30ng/mL; vitamin D deficient group: 25(OH)D < 30ng/mL.

25(OH)D, 25-hydroxy-vitamin D; 1,25(OH)2D, 1,25-dihydroxyvitamin D; iPTH, intact parathyroid hormone; FGF23, Fibroblast Growth Factor 23 receptor; OPG, osteoprotegerin; ALP, alkaline phosphatas.

Coefficients B: unstandardized coefficients B.

95%CI: 95% Confidence interval for B.

Parameters recognized in the literature as affecting PTH concentration (age, serum calcium, serum phosphor, creatinine, FGF23, OPG, ALP, and sclerostin) were introduced into the multivariate linear regression model.

## 4 Discussion

In the current study, we analyzed the potential correlation between two forms of vitamin D (25(OH)D and 1,25(OH)2D) on one hand and three forms of PTH (iPTH, oxPTH and n-oxPTH) on the other hand in 531 transplant patients. The present study revealed a negative comparable correlation between 25(OH)D and all forms of PTH, including oxPTH, the potentially not bioactive or less active form of PTH (**Figure 3A**). No significant correlation was observed between 1,25(OH)2D and all the forms of PTH (**Figure 3B**). Subgroup analysis showed that the above described key findings are not affected by sex and age. OxPTH showed an almost perfect positive correlation with iPTH (r=0.998, *p*<0.0001) (**Figure 2**), however, this positive correlation was less perfect between oxPTH and n-oxPTH (r=0.866, *p*<0.0001) (**Figure 2**).

iPTH is simply the sum of oxPTH and n-oxPTH (29). The perfect correlation between iPTH and oxPTH and the less perfect correlation between iPTH and n-oxPTH might indicate that iPTH is mainly reflecting oxPTH (means the sum of all oxidized forms of PTH) which might be an indicator for oxidative stress rather than biologically active PTH. In agreement with this notion, oxPTH has a substantially lower metabolic clearance rate than n-oxPTH (30, 31). Along with this line, almost 89.48% iPTH was oxidized in the current study. Comparable results regarding the correlations between iPTH, oxPTH and n-oxPTH were also reported in EVOLVE trail—a 2867 participants study (32), and other three observational studies (33–35). Two important remarks would be also relevant in this regard. First, it was reported that PTH oxidation takes place *in vivo*, thus we can exclude the possibility of ex vivo oxidation of PTH due to preanalytical conditions (36). Second, the cohort analyzed in the present study are kidney transplant patients. Their kidney function recovered only partially, they suffer from oxidative stress leading to increased percentage of oxPTH.

The effect of vitamin D supplementation on n-oxPTH concentrations in hypertensive vitamin D-deficient individuals with maintained renal function was studied in a clinical trial– the Styrian Vitamin D Hypertension Trial evaluated 108 vitamin D-deficient hypertensive patients, treated either with vitamin D (2880 IE daily) or placebo for 8 weeks (37). It was shown that both total PTH (tPTH) and n-oxPTH concentrations dropped after supplementation. tPTH decreased more than n-oxPTH. This indicates that vitamin D supplementation increases the non-oxidized fraction of PTH while decreasing the oxidized proportion (37). A challenge here is the lack for approved standard normal values for n-oxPTH by international guideline boards (36). Vitamin D has been shown to have antioxidant properties both *in vitro* and *in vivo*, in rats as well as in humans (38–41). PTH oxidation may thus be influenced by vitamin D supplementation, as current evidence suggests (37). In any case, the finding that both total PTH (tPTH) and n-oxPTH concentrations dropped after supplementation, see above, and the finding in the current study that oxPTH, iPTH and n-oxPTH correlates inversely with 25(OH)D in our study do support the hypothesis that 25(OH)D inhibits the synthesis of all forms of PTH probably already at the level of the parathyroid gland – in other words: 25(OH)D has an negative effect on the synthesis of n-oxPTH and oxPTH in the chief cells of the parathyroid gland.

Low vitamin D status, as well as low dietary intakes of vitamin D and calcium, are considered independent factors increasing PTH levels (42, 43). This present study reveals a strong negative correlation between 25(OH)D and iPTH, which is in line with other studies (43–65). However, our study is the first to show that both bioinactive oxPTH and bioactive n-oxPTH are inversely correlated with 25(OH)D in kidney transplant patients. In this context, it is important to note that oxPTH represents a mixture of oxidized forms of PTH (Met8-oxPTH, Met18-oxPTH and Met8, Met18-dioxPTH). These forms do have different biological properties. Met18 in the PTH molecule is more susceptible to oxidation than Met8 in the PTH molecule (66). Met8 is located in a hydrophobic pocket, which hampers oxidation of this residue as compared to Met(18) (67). Compared to n-oxPTH, PTH molecule with oxidized Met(8) has a less reverse turn structure and more nonordered structure (8). The resulting structural change is still more like n-oxPTH if only Met 18 is oxidized, and thus agrees with the degree of residual biological activity. As of now, the available methods for measuring n-oxPTH cannot differentiate and quantitate the different forms of oxPTH.

Inconsistent results regarding the correlations between 1,25(OH)2D and PTH were reported showing no significant correlation (68), significant positive correlation (58), or significant negative correlation (69, 70). Our current study, when analyzing the whole population, showed clearly that the serum 1,25(OH)2D was associated with iPTH and oxPTH only under the conditions of 25(OH)D≥30ng/ml, and the multivariate linear regression has also confirmed this result. Recently, a large cross-sectional study showed that 25(OH)D and iPTH were inversely correlated across sex and different stages of age but serum 1,25(OH)2D was only positively associated with iPTH in the 25(OH)D<40ng/ml subgroup and in women (71). And in a study of 909 men without known chronic kidney disease and when not taking antidiabetic medications, it was found that a high 1,25(OH)2D/25(OH)D ratio was significantly correlated with higher levels of PTH than the level of 1,25(OH)2D alone (58). There are studies showing that circulating 25(OH)D concentrations in healthy adults have no effect on circulating 1,25(OH)2D (43, 72). Thus circulating 25(OH)D may influence PTH secretion and parathyroid growth *via* 1,25(OH)2D generated within parathyroid chief cells.

Although our study is the first cross-sectional study to simultaneously study the association of 25(OH)D and 1,25(OH)2D with different forms of PTH in renal transplant patients, there are still some limitations. Our study did not compare vitamin D and PTH before and after transplantation due to the lack of such data. We were also uncertain whether the subjects were supplemented with vitamin D or not. The clinical assay used to measure oxPTH and n-oxPTH is only able to differentiate non-oxidized PTH from all forms oxidized PTH (Met8-oxPTH, Met18-oxPTH and Met8, Met18-di-oxPTH). Since Met18-oxPTH is partially bioactive (73), it would have been of interest to measure the oxidized forms of PTH separately. However, this is currently not possible with the methods available for clinical research so far.

## 5 Conclusions

In conclusion, iPTH, oxPTH as well as n-oxPTH are inversely and independent of confounding factors correlated with 25(OH)D but not with1,25(OH)2D. Previous basic science studies have shown that one oxPTH form (Met18-oxPTH) is also bioactive. It is well established that PTH synthesis is blocked in the parathyroid gland by vitamin D. Hence, our finding would be in line with an inhibition of the synthesis of all forms of PTH (bioactive n-oxPTH and oxidized forms of PTH with minor or no bioactivity) in the chief cells of the parathyroid glad.

## Data availability statement

The original contributions presented in the study are included in the article/**Supplementary Material**. Further inquiries can be directed to the corresponding author.

## Ethics statement

The studies involving human participants were reviewed and approved by Charité-Mitte, Berlin, Germany. The patients/participants provided their written informed consent to participate in this study. The study was conducted according to the guidelines of the Declaration of Helsinki, approved by the Ethical Committee of the University of Potsdam (reference number:3a-2013, approval date: February 2, 2014).

## Author contributions

BH conceived the research idea and participated in the writing and revision of the manuscript. JZ contributed to the literature search and data extraction. JZ and AH analyzed the data and wrote the paper. C-FH revised the manuscript. BH, PK, BK, and BKK amended the manuscript. All authors made contributions to the conception and/or implementation of the study, were involved in reviewing and revising the manuscript. All authors contributed to the article and approved the submitted version.

## Supplementary material

Supplementary Figure 1
**(A)** Distribution of 25(OH)D, 1,25(OH)2D. **(B)** Distribution of iPTH, oxPTH, n-oxPTH.

Supplementary Figure 2 Correlation of 25(OH)D and 1,25(OH)2D in 531 kidney transplant patients.

Supplementary Figure 3
**(A)** Relationship between serum 25(OH)D and PTH in females and males. **(B)** Relationship between serum 1,25(OH)2D and PTH in females and males.

Supplementary Figure 4
**(A)** Relationship between serum 25(OH)D and serum PTH in age < 50 years group and age ≥50 years group. **(B)** Relationship between serum 1,25(OH)2D and serum PTH in age < 50 years group and age ≥50 years group.

Supplementary Figure 5 Relationship between serum 1,25(OH)2D and serum PTH (iPTH, oxPTH and n-oxPTH) in 25(OH)D< 30 ng/mL and 25(OH)D ≥30 ng/mL group.
