# Supplementary figures and images for: Inverse correlation of intact PTH, oxidized PTH as well as non-oxidized PTH with 25-hydroxyvitamin D3 in kidney transplant recipients
Source: Front Endocrinol (Lausanne). 2023 May 31;14:1178166. doi: 10.3389/fendo.2023.1178166 (PMC10264784; doi:10.3389/fendo.2023.1178166)

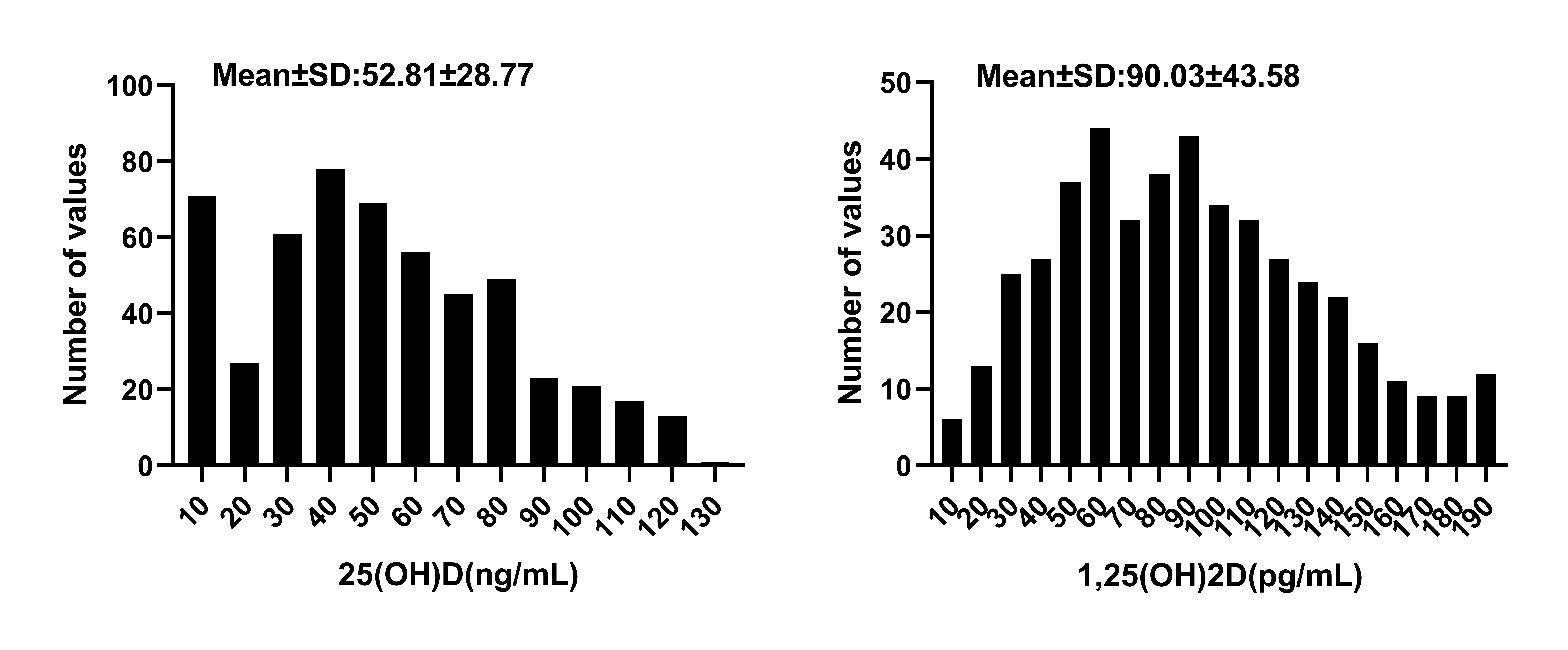

Supplement: Supplementary Figure 1 — (A) Distribution of 25(OH)D, 1,25(OH)2D. (B) Distribution of iPTH, oxPTH, n-oxPTH. [file DataSheet_1.zip › Supplementary Figures/Supplementary Figure 1A.TIF]

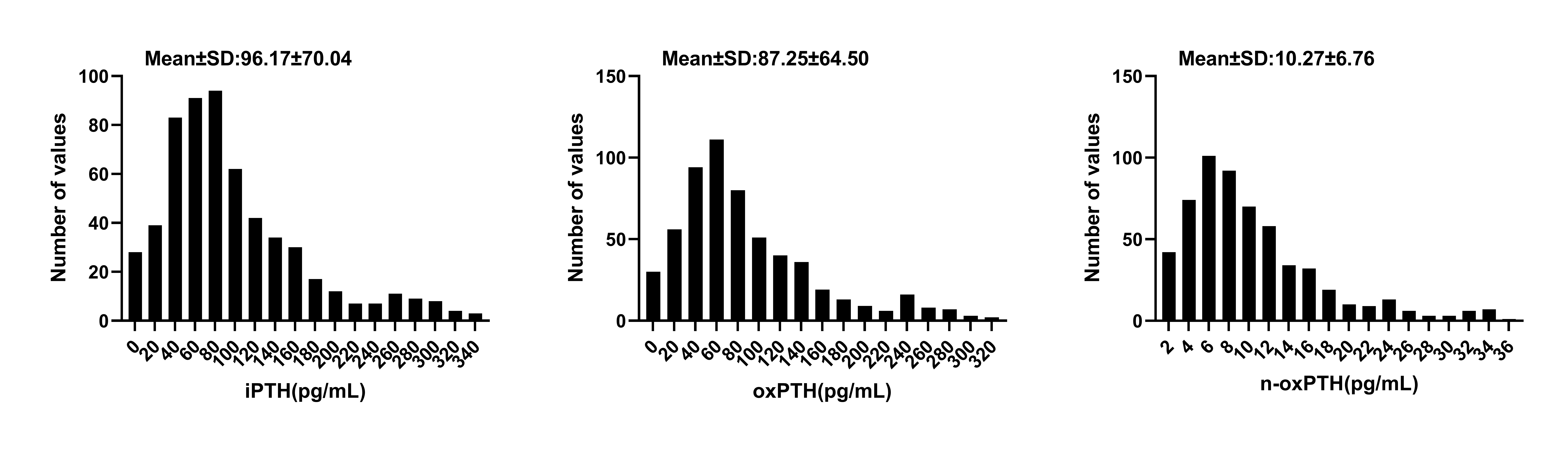

Supplement: Supplementary Figure 1 — (A) Distribution of 25(OH)D, 1,25(OH)2D. (B) Distribution of iPTH, oxPTH, n-oxPTH. [file DataSheet_1.zip › Supplementary Figures/Supplementary Figure 1B.TIF]

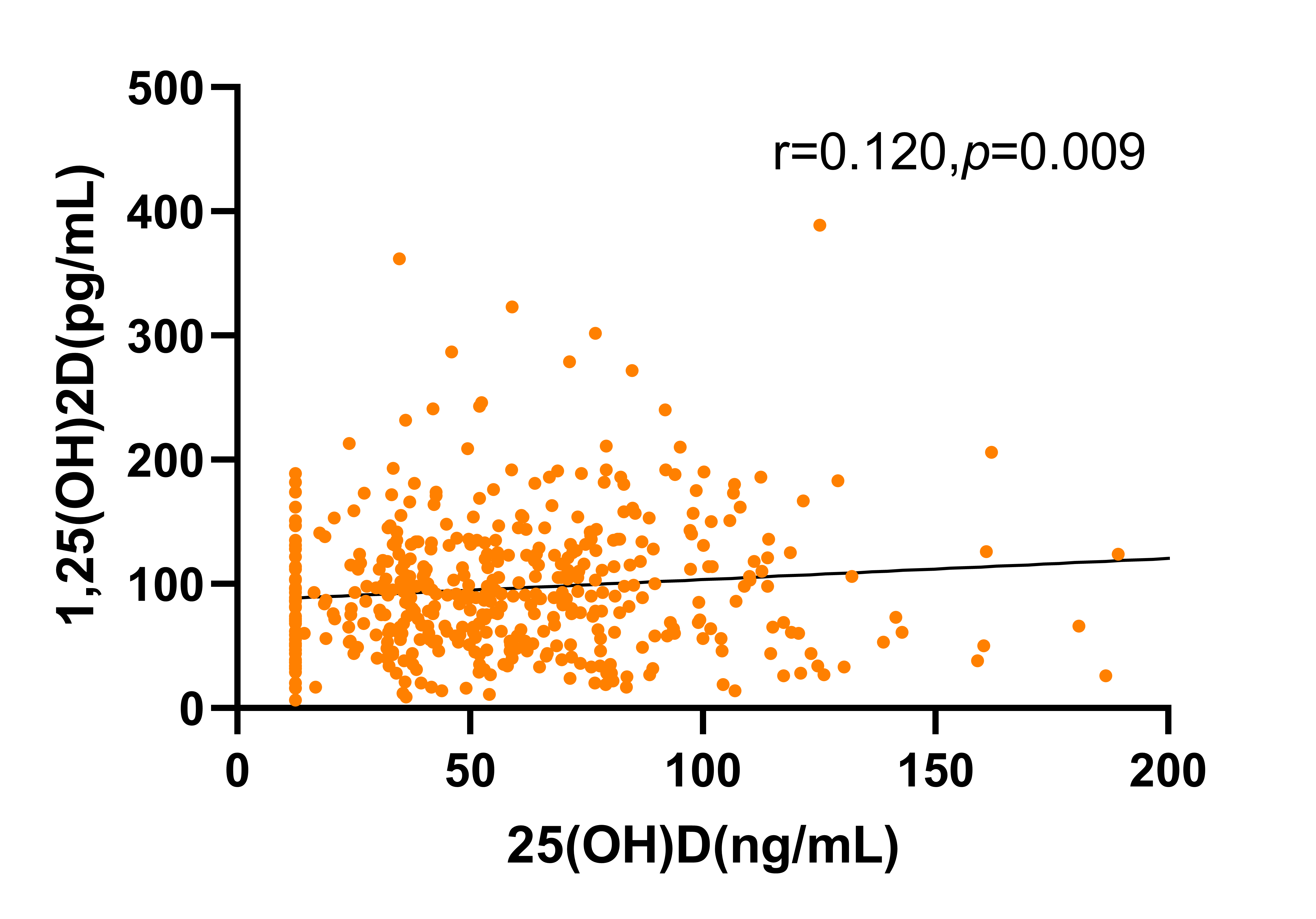

Supplement: Supplementary Figure 1 — (A) Distribution of 25(OH)D, 1,25(OH)2D. (B) Distribution of iPTH, oxPTH, n-oxPTH. [file DataSheet_1.zip › Supplementary Figures/Supplementary Figure 2.TIF]

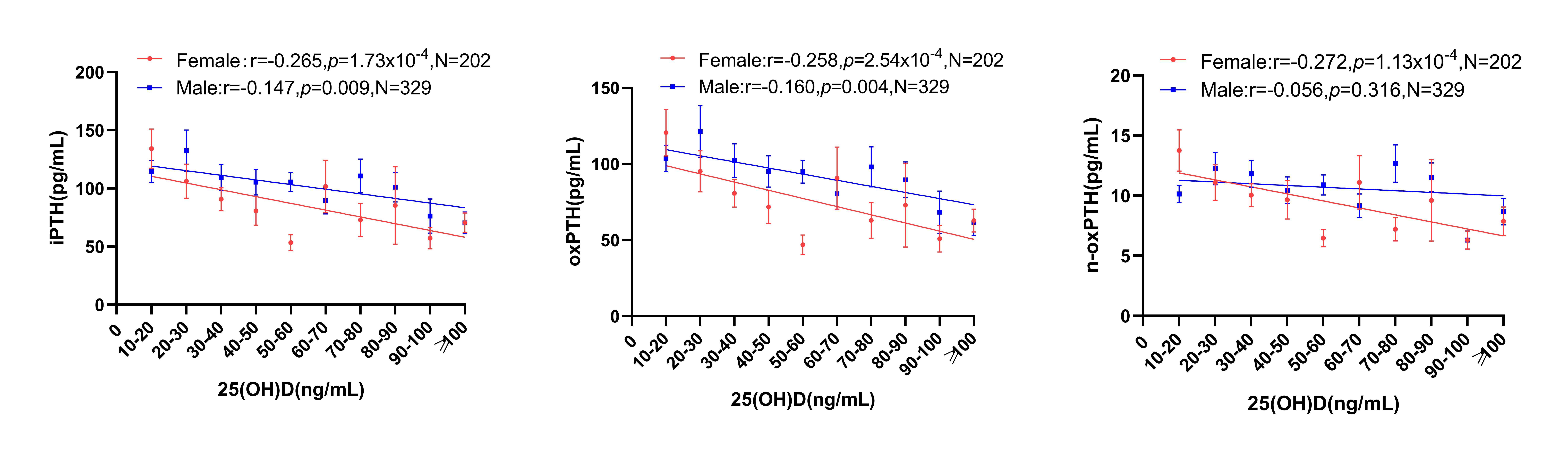

Supplement: Supplementary Figure 1 — (A) Distribution of 25(OH)D, 1,25(OH)2D. (B) Distribution of iPTH, oxPTH, n-oxPTH. [file DataSheet_1.zip › Supplementary Figures/Supplementary Figure 3A.TIF]

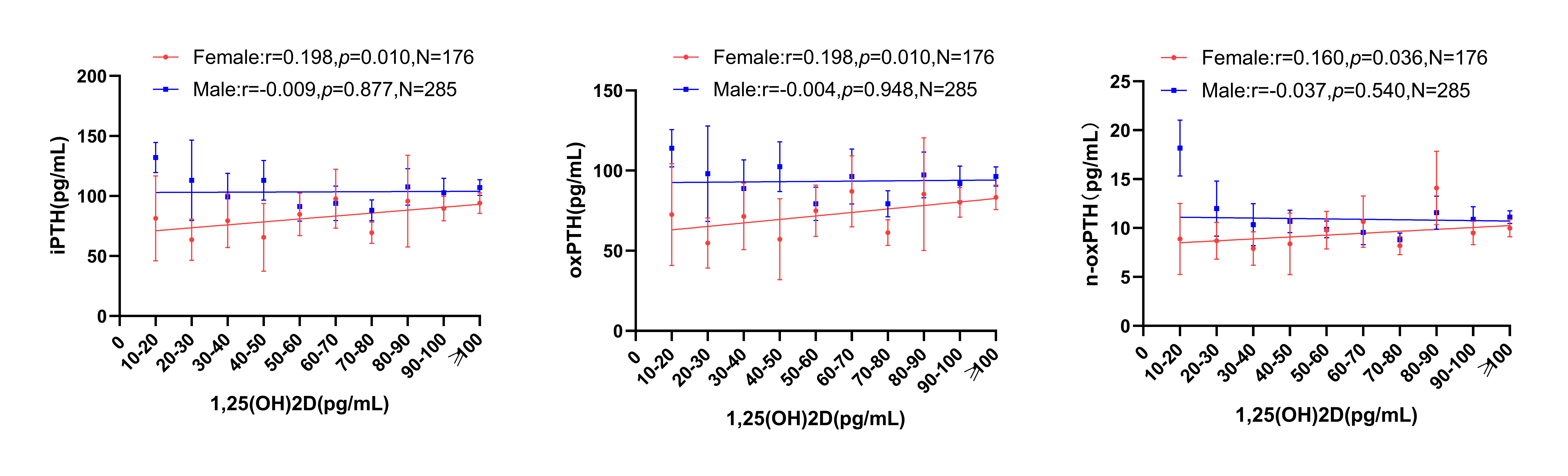

Supplement: Supplementary Figure 1 — (A) Distribution of 25(OH)D, 1,25(OH)2D. (B) Distribution of iPTH, oxPTH, n-oxPTH. [file DataSheet_1.zip › Supplementary Figures/Supplementary Figure 3B.TIF]

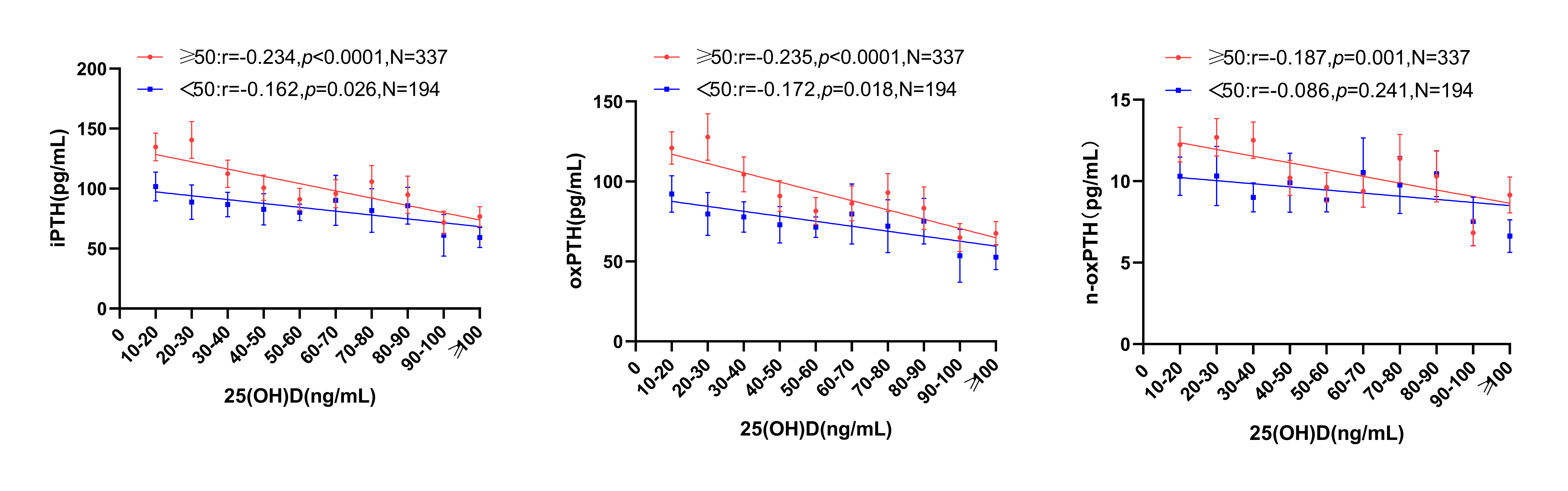

Supplement: Supplementary Figure 1 — (A) Distribution of 25(OH)D, 1,25(OH)2D. (B) Distribution of iPTH, oxPTH, n-oxPTH. [file DataSheet_1.zip › Supplementary Figures/Supplementary Figure 4A.TIF]

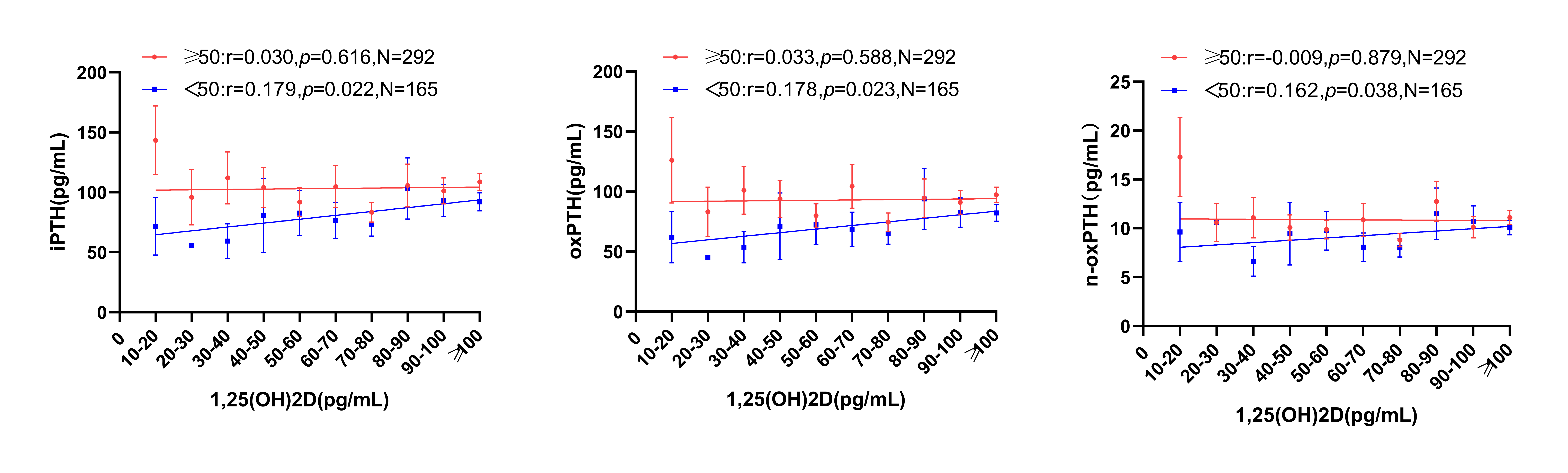

Supplement: Supplementary Figure 1 — (A) Distribution of 25(OH)D, 1,25(OH)2D. (B) Distribution of iPTH, oxPTH, n-oxPTH. [file DataSheet_1.zip › Supplementary Figures/Supplementary Figure 4B.TIF]

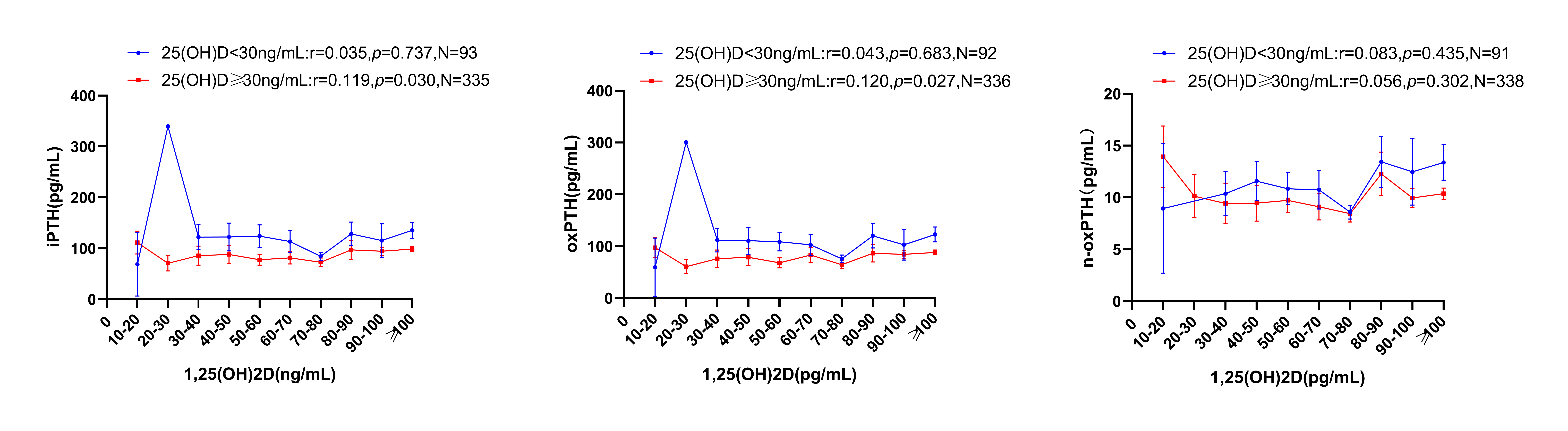

Supplement: Supplementary Figure 1 — (A) Distribution of 25(OH)D, 1,25(OH)2D. (B) Distribution of iPTH, oxPTH, n-oxPTH. [file DataSheet_1.zip › Supplementary Figures/Supplementary Figure 5.TIF]
